# After stroke bidirectional modulation of soleus stretch reflex amplitude emerges during rhythmic arm cycling

**DOI:** 10.3389/fnhum.2014.00136

**Published:** 2014-03-11

**Authors:** Rinaldo A. Mezzarane, Tsuyoshi Nakajima, E. P. Zehr

**Affiliations:** ^1^Laboratory of Signal Processing and Motor Control, University of Brasïlia, College of Physical EducationBrasïlia, Brazil; ^2^Rehabilitation Neuroscience Laboratory, School of Exercise Science, Physical, and Health Education, University of VictoriaVictoria, BC, Canada; ^3^Department of Integrative Physiology, Kyorin University School of MedicineMitaka, Tokyo, Japan; ^4^Human Discovery Science, International Collaboration on Repair DiscoveriesVancouver, BC, Canada; ^5^Centre for Biomedical Research, University of VictoriaVictoria, BC, Canada; ^6^Division of Medical Sciences, University of VictoriaBC, Canada

**Keywords:** locomotion, spinal cord, homosynaptic depression, presynaptic inhibition, spasticity, hyperreflexia

## Abstract

Objectives: after stroke a typical presentation is exaggerated stretch reflexes (SRs) on the more affected (MA) side. The present study evaluated the contribution of presynaptic inhibition (PSI) induced by arm cycling and homosynaptic depression (HD) to the modulation of hyperreflexia at the ankle after stroke. Possible asymmetry of these effects between the MA and less affected (LA) legs was also assessed. Methods: soleus SR was conditioned by: arm cycling at 1 Hz (to increase Ia PSI); or, a preceding conditioning tendon tap applied 1 s before the test stimulus (to induce HD). The extent of conditioning effects was compared between the MA and the LA legs. Results: for both MA and LA legs, rhythmic arm movement induced a bidirectional effect in different participants, either increasing or decreasing SR amplitude (*p *< 0.05). HD had a significant effect in both legs (*p *< 0.05), however, the effect of both a previous muscle stretch and arm cycling was not different between the MA and the LA legs. Conclusion: our data reveal a bidirectional reflex modulation induced by arm cycling that produced facilitation in some and suppression in other participants after stroke. Relative SR amplitude modulation did not differ between the LA and MA legs. We speculate that alterations in SR amplitude modulation after stroke may reflect specific changes in both presynaptic afferent transmission mechanisms and fusimotor control. Significance: the present findings open new perspectives on the characterization of pathophysiology of stroke during the performance of functionally relevant motor tasks.

## INTRODUCTION

Earlier investigations on H-reflex modulation during rhythmic arm or leg movement have provided compelling evidence of the existence of neuronal linkage incorporated in the coordination of all four limbs during locomotion (Dietz, 2002; Mezzarane et al., 2011a). Group I a presynaptic inhibition (PSI) seems to be an important mechanism for reflex modulation in the legs and arms in response to arm and leg cycling, respectively (Frigon et al., 2004; Nakajima et al., 2013).

A previous study of the effect of arm cycling on hyperactive soleus (SO) H-reflexes showed that a portion of the suppression of H-reflex amplitude was maintained after stroke, but was weaker than that seen in neurologically intact participants (Barzi and Zehr, 2008). In that study H-reflex recruitment curves obtained during arm cycling revealed amplitude suppression only for higher threshold motor units (i.e., the largest amplitude H-reflexes) in the more affected (MA) leg. In the less affected (LA) leg both higher and lower threshold motor units were significantly modulated (Barzi and Zehr, 2008).

Hyperreflexia as measured by stretch reflexes (SRs) has been suggested to be closer to the clinical manifestation of spasticity than is the electrically evoked H-reflex (Grey et al., 2008). Essentially nothing is known about modulation of the SR during rhythmic arm movement after stroke. Despite the differences in synaptic transmission between electrically and mechanically elicited reflexes (Burke et al., 1983), the SR might be more susceptible to gamma activity than the H-reflex, as the last one bypasses muscle spindles. Thus, studies with SR provide an opportunity to investigate possible alterations in the fusimotor system. In this context, the first objective of the present study is to investigate the remote effects of arm cycling on the excitability of SR pathway in those with hyperreflexia after stroke. Apart from improving the understanding of neurophysiology, one interesting outcome of the present research is the development of protocols that might help in the identification of specific spinal cord mechanisms responsible for motor impairment. Moreover, a protocol based on a relatively simple procedure (a tendon tap) might be useful for researchers and clinicians engaged in the conception and/or enhancement of rehabilitative procedures.

Several inhibitory pathways are involved in reflex modulation, and their excitability can be investigated through the use of methodologies based on conditioned reflexes (Frigon et al., 2004; Pierrot-Deseilligny and Burke, 2005; Mezzarane et al., 2012). Changes in descending drive strongly affect these pathways and alterations in their excitability constitute important elements to better understand the pathophysiology of stroke. For instance, the PSI onto Ia terminals is reduced in the muscles of the arms and legs on the MA side after stroke (Lamy et al., 2009). Based on this observation, we hypothesized that arm cycling may affect the modulation of the SR in the MA leg less than in the LA one. This finding could argue in favor of an asymmetric presynaptic regulation of reflex excitability in the legs during rhythmic arm movement in the stroke population.

Additionally, evidence suggests that homosynaptic depression (HD) is a mechanism underlying the reflex hyperexcitability seen in spasticity (Lamy et al., 2009). HD of both H-reflex and SR amplitudes in response to previous stretch of the SO muscle have been compared between neurologically intact and participants with spasticity arising from multiple sclerosis or spinal cord injury (Nielsen et al., 1995; Grey et al., 2008). The reduced depression for both reflexes in spasticity suggests that mechanisms additional to PSI contribute to exaggerated excitability of SR pathway. Here we examined the contribution of two different presynaptic mechanisms, namely Ia PSI induced by arm cycling (Frigon et al., 2004) and HD (Hultborn et al., 1996; Kohn et al., 1997), by comparing the modulation of SR excitability in the MA and LA legs after stroke. Therefore, the second objective of the present research is to reveal bilateral differences in transmission at the Ia-alpha motoneuron synapse and functional movement-related presynaptic regulation. Portions of this work have been published in abstract form (Mezzarane et al., 2011b).

## MATERIALS AND METHODS

### SUBJECTS

Twenty participants with chronic stroke (aged 65.9 ± 10.6 years, mean ± SD) volunteered for the present investigation (**Table 1**). A written informed consent approved by Human Research Ethics Committee at the University of Victoria was signed by all participants according to the guidelines in the Declaration of Helsinki. All participants allowed access to their medical records related to the cerebrovascular accident. No participant has his/hers medication suspended by the time of the experiments.

**Table 1:** Participant data and clinical assessment parameters.

Subject	Sex/Age/MA	Lesion location (CT Scan)/Side	MAS (UE/LE)	Brunnstrom (Arm/Leg/Hand/Foot)	FAC	10 m walk (seconds/no of steps)	Time (years)
1	F/64/R	Frontoparietal/B	2/1	3/5/2/2	4	9.44/21	16
2	M/68/L	CR; IC/R	0/0	-/6/7/6	5	5.54/12	1
3	M/48/L	MCA/R	1+/0	3/6/2/2	5	6.18/14	14
4	M/73/L	NA	1/3	2/4/1/2	4	23.84/38.5	24
5	M/58/R	Carotid artery/L	2/1+	4/4/5/2	4	15.22/21	3
6	M/73/R	NA	2/1	3/6/3/6	5	9.44/20	3
7	F/54/L	BG/R	3/0	3/6/2/6	4	7.66/15	2
8	M/70/R	Carotid artery/B	0/0	7/7/7/7	5	6.47/12.5	1
9	F/56/L	Frontal/R	0/0	6/7/6/6	5	6.84/15	32
10	M/81/L	CR; frontoparietal/B	0/0	3/5/6/6	5	8.84/16.25	0.5
11	M/73/L	Brainstem/R	1/1	5/6/7/7	4	9.59/16	40
12	M/73/R	Parietal/L	3/1	2/4/5/1	4	69/56	1
13	F/81/R	Parietal/B	1/0	3/7/2/6	4	8.65/17	3
14	M/67/R	NA	2/1	3/-/3/7	4	6.19/13	1
15	M/44/R	Brainstem/L	2/3	3/2/5/3	1	–/–	15
16	M/61/L	MCA/R	2/0	5/6/4/5	4	12.97/20.5	2
17	M/77/L	IC/R	1/1	6/7/6/6	4	6.22/13	3
18	M/69/L	NA	0/0	5/6/6/6	4	9.38/17.5	8
19	M/74/R	Parietal/L	4/3	1/3/1/2	3	67.9/31	13
20	F/55/L	BG; CR/R	3/1	3/6/3/3	5	9.22/15.5	2

*
MA, more affected leg; R, right leg; L, left leg; B, bilateral; MAS, modified Ashworth scale; UE, upper extremity; LE, lower extremity; FAC, functional ambulation category; time, time after the stroke event; BG, basal ganglia; CR, corona radiata; IC, internal capsule; MCA, middle cerebral artery; NA, data not available.*

Subjects were comfortably seated in a wheelchair with knee and ankle angles at approximately 120° and 110°, respectively, with the feet fixed in a rigid structure. They were instructed to remain relaxed (no muscle contraction). Subjects were asked to grasp the handle of an arm cycling ergometer (described in Zehr et al., 2004). A custom made wrist brace was used in the MA arm to assist with the grip during arm cycling. The length of the crank was adjusted for each participant to accommodate the limited range of motion of the MA arm.

### CLINICAL ASSESSMENT

All participants underwent a clinical assessment within a few days of the main experiments (details in **Table 1**). The severity of spasticity in the arms and legs was assessed by the modified Ashworth scale (MAS; Bohannon and Smith, 1987; Lee et al., 1989; Blackburn et al., 2002). For correlation procedures, the “1+” values displayed in **Table 1** were replaced with “1.5.” Seven stages of motor recovery (where 1 represents flaccid paralysis and 7 is normal) were estimated in the MA side at the hand, arm, foot and leg through the Brunnstrom score (Brunnstrom, 1966).

A locomotor scale (functional ambulation category, FAC) with good inter-rater reliability (Holden et al., 1984) was used to rate the dependence on external aid (devices or another person) to assist with locomotion on level surfaces. Higher scores indicate higher independence (Holden et al., 1984). Other measurements such as period of time and number of steps needed to walk a distance of 10 m (Collen et al., 1990) were also performed (see **Table 1**).

### ELECTROMYOGRAPHY

Surface electromyogram (EMG) was recorded bilaterally from five muscles: anterior deltoid (AD), biceps femoris (BF), vastus lateralis (VL), SO, and tibialis anterior (TA) using disposable 1 cm Ag–AgCl surface electrodes (Thought Technologies, Edmonton, AB, Canada) with inter-electrode distance of 2 cm. The ground electrode was placed on bony landmarks near the target muscle. The skin over the belly of each muscle was prepared by using alcohol swabs.

EMG signals were sampled at 2.5 kHz with a 12-bit A/D converter connected to a computer running custom-written Lab View software (National Instruments, Austin, TX, USA). The signals were amplified (5000 times) and band-pass filtered (100–300 Hz) by a Grass P511 amplifier (Grass Instruments, AstroMed). When the SO muscle was used to evoke a SR or a *M*_max_ (see Stimulation) the band-pass filter was set at 100–1000 Hz (with gain of the amplifier at 1000). The sweeps of EMG were 100 ms duration with a 20 ms prestimulus period. For the double pulse paradigm (to obtain HD) the sweep total duration was 1120 ms, with prestimulus period of 20 ms for both responses.

### STIMULATION

As used previously (Palomino et al., 2011; Mezzarane et al., 2012), an electrodynamic vibration system (“Shaker”; model LW-126-13, Labworks) was positioned behind the foot and a slight pressure of the shaker tip was applied against the Achilles tendon. This pressure was maintained constant for all subjects and was set based on how much (3 mm) the main movable cylinder (to which the tip of the shaker was attached) came inside the external shield of the device. The parameters of mechanical stimulus was defined in a Lab View custom-written program (National Instruments) as being a single sinusoidal cycle with 100 Hz (10 ms duration) fed into the shaker controller. The amplitude of the shaker tip excursion was defined directly in the controller during the experiment, after setting a reference gain in the Lab View window. An accelerometer (ADXL193; Analog devices) was fixed at the tip of the shaker to provide the displacement (Fornari and Kohn, 2008; Mezzarane et al., 2012).

Before the experiment, three to five maximal direct muscle responses (*M*_max_) were obtained from the SO muscle of both limbs in 18 out of 20 subjects by applying percutaneous supramaximal electrical stimulation (square wave pulse of 1 ms duration) to the posterior tibial nerve in the popliteal fossa. Bipolar surface electrodes were used according to established protocols and methodologies (Zehr, 2002). A constant current stimulator (Digitimer Ltd. UK; model DS7A) was used to deliver the stimulus.

### PROTOCOL

**FIGURE 1:**
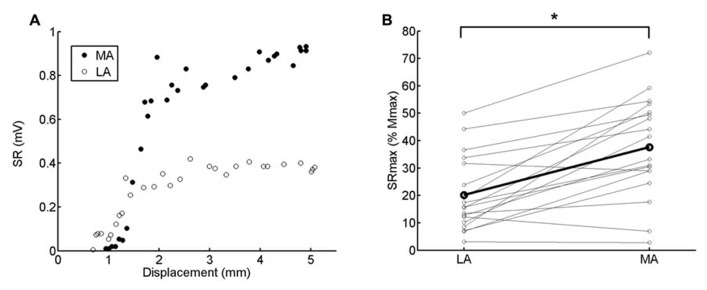
**(A)** Stretch reflex (SR) recruitment curve obtained in both legs from one representative subject (<3). The displacement of the shaker tip is displayed in the abscissa. The SR_max_ was calculated from the average of the 3 highest amplitude values. Higher absolute values are clearly observed for the MA compared to the LA leg. **(B)** Pairwise comparison of SR_max_ (normalized by *M*_max_) between LA and MA legs. The thicker black line connects the averaged responses calculated from 18 subjects. Asterisk indicates significant difference (*p *< 0.0001).

Stretch reflex recruitment curves (*n* = 30 points) were obtained before the beginning of the experiment with the subject in a static position by randomly varying the intensity of the mechanical stimulation (**Figure 1A**).

The test SR amplitudes for both legs ranged between 50% and 70% of the corresponding maximal SR (SR_max_). While we use both normalization to *M*_max_ and SR_max_ for reference, there are a number of reasons for preferring SR_max_ for normalization. We were concerned *a priori* that we might not be able to obtain *M*_max_ in all subjects. Also, we wanted to avoid the use of too low test reflex amplitudes, i.e., **Figure 1B** shows that few subjects had their SR_max_ values lower than 10% *M*_max_. In this case, we would have to choose test amplitude at around 3–7% *M*_max_ to include all available subjects in the analysis. Furthermore, with the current procedure a wider range of motor unit sizes was covered allowing a more suitable comparison with previous findings using the H-reflex in the stroke population (Barzi and Zehr, 2008). Lastly, we wanted to use methodologies that could be widely incorporated in the stroke population. In this clinical group allodynia is not uncommon and discomfort associated with evoking maximal M waves occurs. As a result, it is not commonly possible to comfortably obtain *M*_max_ and, therefore, calibration with another reference point is necessary.

#### Conditioning SR

Participants performed arm cycling on a customized ergometer (Zehr et al., 2004; Barzi and Zehr, 2008) at ~1 Hz with the aid of visual feedback. For reference, the movement cycle was divided into 12 parts corresponding to hours on a clock face. All reflex responses were evoked at the position “7 o’clock,” which corresponded to nearly the middle way of the movement of pulling the hand back to the body from a full extension of the elbow (3 o’clock) to a full flexion (9 o’clock) and was previously shown to have the largest H-reflex suppression (de Ruiter et al., 2010). The right arm was used as a reference for all participants (as in Barzi and Zehr, 2008), irrespective of their affected side, since reflex suppression was also observed in the contralateral leg to the cycling movement (Loadman and Zehr, 2007). Any possible differential effect from the reference arm positioning (ipsi or contralateral to the leg under study) was minimized by the fact that the occurrence of MA limb was almost equally distributed across the participants (**Table 1**).

The SR was elicited at rest, while holding the ergometer crank at the 7 o’clock position, and during cycling. The SR was evoked at random intervals between 3 and 5 s and 15 reflexes were obtained in each condition. This same procedure was then repeated for the contralateral leg by repositioning the shaker. Each procedure lasted about 2 min and rest periods of 5 min between them were allowed.

For the study of HD, both single and double pulse conditioning were randomly presented with intervals between them varying from 8 to 12 s. The single pulse was used to minimize any predictive effects on the reflex amplitudes evoked by the second pulse. Twenty (10 single and 10 double) sweeps were obtained from each leg. The duration of each procedure was around 4 min.

### DATA PROCESSING

All data were analyzed off-line by using custom-written Matlab routines (MathWorks). Peak-to-peak amplitudes of SRs and *M*_max_ along with the root mean square (RMS) values of the background EMG (bEMG; corresponding to 20 ms prestimulus period) were calculated.

The SR amplitudes obtained in all conditions were normalized to SR_max_. For the double pulse experiment (to induce HD of the SR) we defined SR1 as the reflex response evoked by the first pulse of the pair, and SR2 the response evoked by the second one (delivered 1 s later). The ratios SR2/SR1 and cycling/static were used in the correlations with clinical parameters.

The maximal reflex response (SR_max_) was calculated as the average of the three highest reflex amplitudes from the recruitment curve, obtained in the static condition before the beginning of the experiments (**Figure 1A**). In order to examine potential asymmetry of reflex hyperexcitability between both legs, the maximum obtainable reflex output (SR_max_) was normalized by the respective *M*_max_ (**Figure 1B**).

**FIGURE 2:**
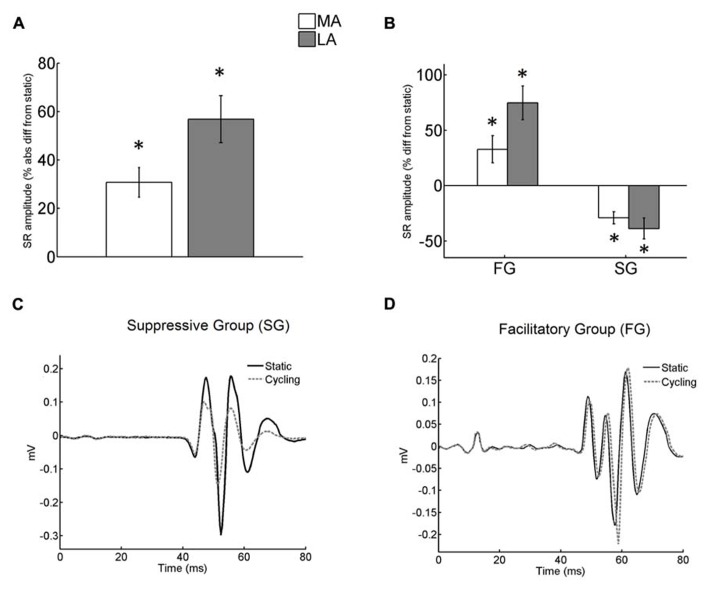
**(A)** Absolute difference (in %) of the stretch reflex (SR) conditioned by arm cycling from SR in static condition. The difference from static was significant for both legs (asterisks mean *p *< 0.001). No significant difference of arm cycling effect was detected between legs; **(B)** difference of conditioned SR from static (in %) for both FG and SG groups. The difference from static was significant for both legs and both groups (asterisks mean *p *< 0.001 and *p *< 0.003 for FG and SG, respectively). However the effect of arm cycling between legs was not different for both groups; **(C)** Individual recordings obtained in static and cycling conditions from the MA leg. Each sweep is an average from a total of 15. Sweeps obtained during static and cycling conditions from subject <3 belonging to Suppressive Group (SG). Note the decreased reflex amplitude during cycling. **(D)** Reflex response (mean of 15 traces) from a representative subject (<14) of Facilitatory Group (FG). There was an increase in reflex amplitude during cycling. The zero value in the abscissa means the instant of stimulus delivery.

The absolute modulation of arm cycling on SR amplitude from static control was evaluated and expressed as percentages. We then grouped together the subjects who showed suppressed conditioned SR (suppressive group, SG) and those that showed facilitatory effect (facilitatory group, FG) for each leg (examples from representative subjects are depicted in **Figure 2**). The main purpose of this analytical procedure was to describe differential effects (asymmetries) from arm cycling between the legs. The same procedure has been described to compare both reflex suppression and facilitation induced by arm cycling in TA muscle (Dragert and Zehr, 2009) and to compare H-reflexes that increased or decreased in response to visual manipulation (Tahayori et al., 2012).

The peak-to-peak value from the accelerometer signal was obtained based upon the sinusoidal displacement of the shaker tip. The displacement was calculated as:

$$D=\frac{ga}{2\pi^{2}F^{2}},$$

In which “*D*” is the displacement of the tip of the shaker in contact with the tendon, “*g*” is the acceleration due to gravity, “*a*” is the acceleration measured by the accelerometer, and “*F*” is the frequency of the sinusoid (100 Hz).

### STATISTICS

A two-way ANOVA was employed to detect differences in the normalized SR values between conditions (static versus cycling, or SR1 versus SR2), legs (LA versus MA) and interactions between factors (condition versus leg).

Student’s paired *t*-test was employed to detect differences in the absolute change from static between legs (LA and MA), shaker tip displacement and bEMG between conditions (static versus cycling). One-sample *t*-test was used to verify if the absolute difference of conditioned SR from static condition was significant (general arm cycling effect).

Pearson product–moment correlations (*r*) were calculated between the eight clinical parameters (except for FAC; **Table 1**) and the four reflex parameters (ratios cycling/static of SG and FG, SR1/SR2 and SR_max_/*M*_max_) for each leg. The clinical parameter FAC was not considered in the correlations as two subjects were distributed across two classes, 1 and 3 (i.e., only one subject per class; **Table 1**), and the contribution of their values would be overestimated. Only the statistically significant correlations were reported in the section “Results.” Pearson’s *r*-values were estimated for the comparison between reflex parameters and clinical assessment measurements such as time and number of steps needed to walk a distance of 10 m (**Table 1**). Correlations between reflex parameters and rank-based clinical assessments (MAS and Brunnstrom score; **Table 1**) were assessed by Spearman’s rank order correlation (*rho*). Descriptive statistics included mean and standard error of mean (SEM). Statistical package SPSS (V.16.0) was used to perform the analysis. Differences were considered significant at *p* < 0.05 for all tests.

## RESULTS

### SR CHARACTERISTICS

Exaggerated SR amplitude is clearly seen in the MA as compared to LA leg in the single subject recruitment curve (static condition) depicted in **Figure 1A**. This was observed for the group data as well. There was a significant difference in the SR_max_ normalized by *M*_max_ between MA and LA legs for all subjects (**Figure 1B**; *p *< 0.0001). The SR_max_ of the MA leg was, on average, 90% higher than LA leg (LA: ~20% *M*_max_; MA: ~40% *M*_max_), indicating hyperreflexia in the MA leg (**Figure 1B**).

### CONDITIONING EFFECT OF ARM CYCLING

Unlike the observations for H-reflex modulation in stroke (Barzi and Zehr, 2008) but similar to the results in TA muscle for neurologically intact participants (Dragert and Zehr, 2009) effects of arm cycling yielded bidirectionally conditioned SRs with amplitude values that were either substantially higher or lower than the control (static) values (**Figures 2A,B**). A significant mixed effect from arm cycling for both legs (*p *< 0.001) was detected, but this effect was not different between legs (*t*-test with Bonferroni correction; **Figure 2A**).

Nearly half of the subjects showed suppression (SG) of SR amplitude for both the LA (*n* = 10) and the MA (*n* = 11) leg, while the other half showed a facilitation (FG; LA: *n* = 10; MA: *n* = 9; **Figure 2B**). **Figures 2C,D** show examples from representative subjects.

In the SG group suppression was ~29% and ~39% for the MA and LA legs, respectively (**Figure 2B**). In the FG, SR reflexes were facilitated ~33% and 75% from static control amplitude in MA and LA legs, respectively (**Figure 2B**). Two-way ANOVA detected significant effects of arm cycling for both FG [*F*_(1,34)_ = 12.8; *p *= 0.001] and SG [*F*_(1,38)_ = 10.2; *p *= 0.003]. However, there was no significant interaction between the legs [FG: *F*_(1,34)_ = 0.91; *p *= 0.35 and SG: *F*_(1,38)_ = 0.34; *p *= 0.57].

No significant correlation was detected between SR conditioned by arm cycling and clinical parameters. Subjects <12 and <19 were considered as outliers for the parameters time and number of steps to cover a distance of 10 m. These subjects took longer than 1 min to perform the task, which is more than 2 SDs from the sample mean.

During arm cycling the SRs from either legs were evoked when the right arm reached the position 7 o’clock. We also examine a possible effect of arm position on the SR amplitude of the MA leg. The absolute change in the conditioned SR amplitude sampled when the MA leg was in the ipsi or contralateral side to the reference arm (right arm at 7 o’clock) were both significant (*p* < 0.01). The effect of arm cycling, however, did not differ bilaterally (results not shown).

### EXTENT OF HOMOSYNAPTIC DEPRESSION

**FIGURE 3:**
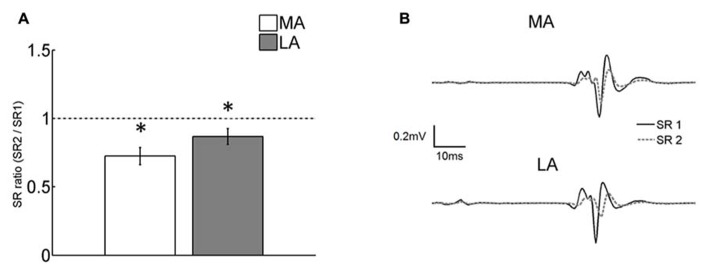
**(A)** Comparison of the ratio of the first (SR1) and the second (SR2) SR evoked in the double pulse paradigm. The second response was significantly (*p *< 0.05) depressed compared to the first one in both the LA and MA legs. The degree of HD was not different between legs. Asterisks indicate significant differences from control (SR1) at *p *< 0.05. Vertical bars indicate SEM; **(B)** averaged sweeps from a representative subject showing SR1 and the depressed SR2.

Homosynaptic depression obtained by evoking a second reflex (SR2) 1 s after the first one (SR1) was, respectively, ~13 and ~28% for the MA and the LA legs. The effect of HD on SR was significant for both legs [*F*_(1,76)_ = 9.75; *p *= 0.002; **Figure 3A**]. The double pulse conditioning showed no significant difference in HD between the legs [no interaction; *F*_(1,76)_ = 1.16; *p *= 0.28]. This means that there is no differential effect of HD. **Figure 3B** shows the averaged SRs from the MA and LA limbs of a representative subject. Only the depressed SR (in response to a previous stretch) showed significant correlation with the MAS. HD was more pronounced for higher values of MAS at the lower extremity (*ρ* = -0.53; *p *= 0.016).

### EXPERIMENTAL CONTROL PARAMETERS

Control reflexes taken in the static condition ranged between 50% and 70% of SR_max_ (see Section “Materials and Methods”) with no significant difference between the LA (59.3 ± 4.0% SR_max_) and MA (55.1 ± 3.2% SR_max_) legs. In the double pulse paradigm, no significant difference in response to the first pulse (SR1) between the LA (59.4 ± 4.2% SR_max_) and MA (53.4 ± 3.8% SR_max_) legs was detected.

Some participants were not able to maintain the quiescent leg muscles throughout the experiment and there were some muscle activation to different extent in cycling conditions. Despite that, bEMG activity of the SO muscle was not different between static and cycling conditions for either the MA or LA legs (results not shown). The same was found for comparison between the bEMG of all muscles recorded 20 ms before the first and the second pulses in the double pulse paradigm (results not shown). This means that the conditioned reflex amplitudes were not affected by possible changes in muscle activation.

The stimulus used to evoke the SR (displacement of shaker tip) was consistently controlled across the experiment. No differences were detected between both static and cycling conditions in both LA and MA legs. Similarly, displacement of the first and the second pulses were not different (not shown). Therefore, observable changes in reflex responses were not due to changes in the mechanical stimulus used to evoke the SR.

## DISCUSSION

The present study confirmed a significant effect of arm cycling on SR reflex amplitude after stroke. Unlike earlier work showing consistent suppression of H-reflex amplitude during arm cycling, SR amplitudes could be either facilitated or suppressed. This difference from static control indicates a bidirectional influence from arm movement. However, the strength of these effects was not different between the legs. There was also no difference between the legs in the extent of HD. Our results suggest that stroke participants have a distinct pattern of reflex response probably related to alterations in neuronal pathways responsible for interlimb coordination.

### POSSIBLE PATHOPHYSIOLOGICAL MECHANISMS

Diversity of pathophysiological alterations can be a result of the lack of specificity of the lesions and the different levels of involvement in these pathways (Calautti and Baron, 2003). Hence, variability observed in the electrophysiological parameters obtained from a stroke population might make it difficult to uncover some important mechanisms underlying motor impairment. The separation into different groups based on the degree of reflex suppression/facilitation would be an alternative to better characterize these mechanisms (Simon, 1996). This procedure might help to evaluate a differential effect of the impaired reflex modulation from descending commands.

Participants with spasticity have difficulty in modulating the excitability of inhibitory pathways in different motor tasks (Morita et al., 2001; Kagamihara and Masakado, 2005; Nielsen et al., 2005; Pierrot-Deseilligny and Burke, 2005). Therefore, when compared to neurologically intact individuals, an altered and possibly differential effect of remote movement conditioning may be expected on presynaptic mechanisms involved in reflex modulation in the legs (Barzi and Zehr, 2008; Zehr and Loadman, 2012). It has been proposed that the activity of cervical spinal cord oscillators related to arm cycling movement increases the level of Ia PSI at Ia terminals in SO (Frigon et al., 2004). This mechanism appears partially preserved after stroke (Barzi and Zehr, 2008) and explains the data obtained from SG.

Results in the FG, however, could be due to the influence of arm cycling being overwhelmed by other factors beyond changes in the level of Ia PSI. One possibility is the change that might occur in the excitability of the fusimotor system (Mazzocchio and Rossi, 1997; Pierrot-Deseilligny and Burke, 2005). It is possible that this group of stroke participants had an abnormal increased level of gamma activity due to the performance of arm cycling, making the SR more responsive (Ribot-Ciscar et al., 2009). The mechanisms responsible for the observed reflex facilitation probably are not related to post-activation depression of Ia-motoneuron synapse (HD), as most of the subjects who showed reflex facilitation in either legs also showed HD in both (10 out of 14). This result along with absence of facilitation in H-reflex amplitude observed in stroke participants during arm cycling (Barzi and Zehr, 2008) reinforces the thesis of a possible contribution of the fusimotor system to the observed SR facilitation.

### SIMILARITY OF CONDITIONING EFFECTS IN THE MA AND LA LEGS

The lack of asymmetry observed for both presynaptic mechanisms is in agreement with previous argument that the LA limb also presents alterations compared to healthy subjects (Thilmann and Fellows, 1991). This contrasts with the asymmetries previously identified using the H-reflex conditioned by arm cycling (Barzi and Zehr, 2008) and by previous reflex activation (Lamy et al., 2009). Interestingly, here the descending effects from cervical spinal centers were examined using a clinically relevant probe (i.e., SR) that showed a bimodal pattern of reflex modulation.

The different results might therefore be related to the type of probe used. That is, reflex modulation associated with the fusimotor system would be more clearly revealed by measurement with SR compared to H-reflex (Rossi-Durand, 2002). However, the afferent volley used as input to evoke both reflexes has distinct characteristics (Burke et al., 1983; Van Boxtel, 1986), and any comparison should be carefully considered. In the H-reflex technique, the electrical stimulation fires a single and synchronized discharge in the afferents from group I, while the mechanical stimulus induces a temporally dispersed burst of discharge of primary muscle spindle endings (Van Boxtel, 1986). This sensory activity may produce a longer rise time of the post-synaptic potential in the motoneurons, which allows the interference of oligosynaptic pathways from different origins (Burke et al., 1983).

To our knowledge, no study has yet been conducted to investigate bilateral HD of the SR in post-stroke hemiparesis. Bilateral symmetry in the degree of HD of the H-reflex was studied in healthy participants (Aymard et al., 2000; Mezzarane and Kohn, 2002). Here we found significant HD of the SR in both legs. Yet, in spite of its suggested importance as a mechanism underlying spasticity (Lamy et al., 2009), we were unable to detect significant differences between legs. Reduced sensitivity of SR to both Ia PSI (Morita et al., 1998) and HD (Grey et al., 2008) compared to the H-reflex could partially mask the pathophysiological-related differences between both limbs. Thus, the use of SR in studies of asymmetries of HD between the MA and the LA legs of stroke participants might be limited.

### TRANSLATIONAL IMPLICATIONS

The present experimental paradigm revealed an unpredictable change in the direction of reflex excitability in response to arm cycling in stroke individuals. This indicates that the post stroke effects extends beyond the observable modulation of reflex responses that bypass muscle spindles, such as the H-reflex. In this respect, the contribution of the fusimotor system needs to be carefully considered in order to understand the nature of the modulation in the SR pathway.

It is conceivable that the unexpected changes in SR amplitude during arm cycling (i.e., reflex facilitation) in post-stroke participants are related to alterations in gamma motoneuronal excitability. This inference could not be made only based on the results obtained from studies of H-reflex modulation during arm cycling (Barzi and Zehr, 2008). It is currently suggested that changes in the fusimotor excitability in stroke need to be considered before recommending interventions based on the use of arm cycling aimed at minimizing hyperreflexia in the spastic legs. An alternative intervention should be prescribed for those patients who present a reflex facilitation (as those belonging to FG in the current study). Other types of rhythmic movements of upper limbs in which the subject is able to perform with less physical effort (e.g., with lower frequency) could be explored as alternatives.

The present experimental paradigm could also be useful to better characterize stroke populations based on the up or down regulation of their reflex excitability during arm cycling. Additional investigations (whether based on SR or not) could further explore other possible patterns and help to elucidate the pathophysiological mechanisms behind reflex modulation after stroke, as well as to develop and refine new tolls as adjunct for rehabilitative procedures.

## AUTHOR CONTRIBUTIONS

Conception and design of the work: Rinaldo A. Mezzarane, Tsuyoshi Nakajima, and E. P. Zehr; Data acquisition: Rinaldo A. Mezzarane and Tsuyoshi Nakajima; Data analysis: Rinaldo A. Mezzarane , Tsuyoshi Nakajima, and E. P. Zehr; Interpretation of the data: Rinaldo A. Mezzarane, Tsuyoshi Nakajima, and E. P. Zehr; Drafted manuscript: Rinaldo A. Mezzarane; Critical review of manuscript: Rinaldo A. Mezzarane, Tsuyoshi Nakajima, and E. P. Zehr; Final approval of the version to be published: Rinaldo A. Mezzarane, Tsuyoshi Nakajima, and E. P. Zehr; Agreement to be accountable for all aspects of the work in ensuring that questions related to the accuracy or integrity of any part of the work are appropriately investigated and resolved: Rinaldo A. Mezzarane, Tsuyoshi Nakajima, and E. P. Zehr.

## Conflict of Interest Statement

The authors declare that the research was conducted in the absence of any commercial or financial relationships that could be construed as a potential conflict of interest.
